# Preliminary Experiences With Robot-Assisted Choledochal Cyst Excision Using the Da Vinci Surgical System in Children Below the Age of One

**DOI:** 10.3389/fped.2021.741098

**Published:** 2021-09-23

**Authors:** Xiaolong Xie, Yang Wu, Kewei Li, Chengbo Ai, Qi Wang, Chuan Wang, Jing Chen, Bo Xiang

**Affiliations:** Department of Pediatric Surgery, West China Hospital, Sichuan University, Chengdu, China

**Keywords:** robot, choledochal cyst excision, da Vinci, children, 1 year old

## Abstract

The purpose of this study is to introduce our preliminary experiences with using the da Vinci surgical system to treat choledochal cysts in children under 1 year old and discuss the application of this robot-assisted surgery. We retrospectively analyzed all available clinical data of children below the age of 1 who underwent surgery for choledochal cysts using the da Vinci robotic surgical system between January 2015 and December 2020. Data collection mainly included demographic information, imaging data, perioperative details, and postoperative outcomes. Ten patients were included in this study. The average patient age was 8.5 months, and the average weight was 9.11 kg. Half of these patients suffered from abdominal pain, while 30% exhibited vomiting and 10% jaundice. Eight of them were type Ia, and two were Ic. The average operation time among the patients was 219.5 min. None of the 10 patients had to receive a blood transfusion or conversion. The average time of the patients' subsequent fluid diet was 3.28 days, and the solid diet was 3.76 days. Meanwhile, the average length of hospital stay was 7.6 days. All 10 patients recovered and were eventually discharged. We believe that the da Vinci surgical system is a safe and feasible form of treatment for choledochal cysts in children <1 year old.

## Introduction

Choledochal cysts are the most common congenital malformation found in the biliary tract and are characterized by cystic or fusiform dilatation of the common bile duct. These have also been known to simultaneously appear alongside intrahepatic bile duct dilatation (1–3). Without effective treatment, patients with choledochal cysts may suffer from cyst perforation, recurrent pancreatitis, cancer, or even severe cholestasis, which can then lead to liver cirrhosis, portal hypertension, and eventually liver failure (4).

The best option for treatment of choledochal cysts is surgery, which mainly involves choledochal cyst resection, cholecystectomy, and hepaticojejunostomy (5). Minimally invasive approaches toward choledochal cysts are currently the mainstream method, and this includes both laparoscope-assisted and robot-assisted surgeries. However, the laparoscopic procedure is as of yet not widely promoted throughout the world because a laparoscopic choledochojejunostomy is technically demanding, as well as requiring a certain learning curve. Therefore, robotic procedures are usually proposed an alternative form of minimally invasive surgery on choledochal cysts due to their unique three-dimensional (3D) imaging and the flexible design of their simulation manipulator, which significantly improve operability and accuracy (6). Woo et al. reported the first robot-assisted choledochal cyst resection in children and their success with it in 2006 (7). The technology has been reported time and time again since then, with several other successful operations taking place (6, 8, 9). However, there are still few reports regarding robot-assisted choledochal cyst resection in the under 1-year-old age group available in the literature (8). Our department alone completed 134 operations on choledochal cyst resection using the da Vinci surgical system between January 2015 and December 2020. Of these, 10 cases were in children below the age of 1, and in this study, we present our experiences and discuss the relevant technical points.

## Methods

### Study Population

We retrospectively analyzed clinical data from patients below the age of 1 year who had undergone robot-assisted surgery to treat choledochal cysts from January 2015 to December 2020 in the West China Hospital of Sichuan University. Informed consent was naturally obtained from the children's parents. The study passed the ethics review of our hospital ethics committee (No. 1082). Candidates for inclusion in the study were based on the following requirements: (1) patients were diagnosed with choledochal cysts through preoperative history, physical examination, B-ultrasound, computed tomography (CT), or magnetic resonance cholangiopancreatography (MRCP); (2) patients would be able to tolerate CO_2_ pneumoperitoneum during general anesthesia and robotic surgery; and (3) the patient's coagulation function was normal, and they had no serious organ dysfunction. Conversely, exclusion criteria included secondary operations, cyst perforation, and malignant transformation of the choledochal cyst before the operation.

### Procedure of the Operation

**Figure 1:**
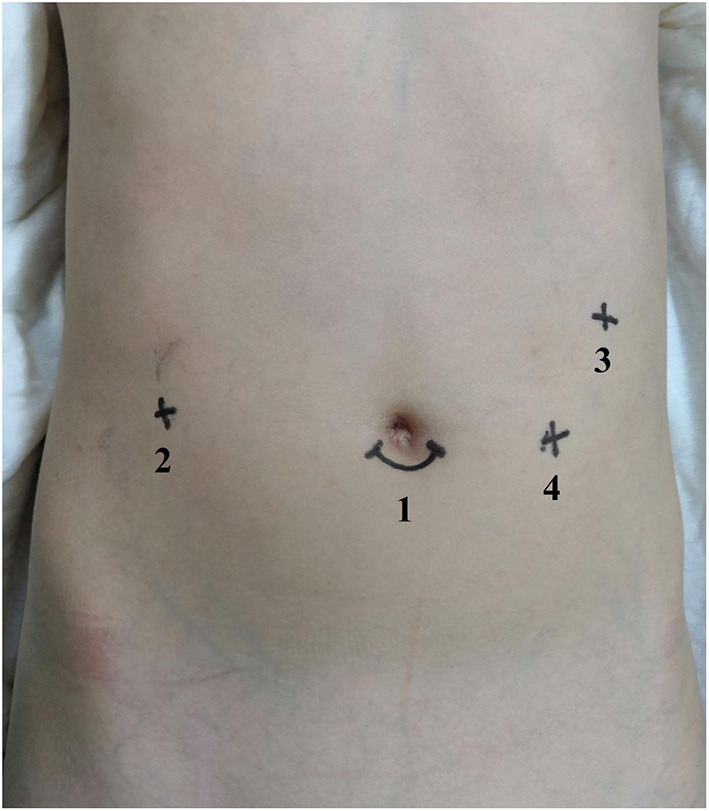
Port placement in robot-assisted surgery for choledochal cysts. (1) Camera port. (2) Port I. (3) Port II. (4) Assistant port.

**Figure 2:**
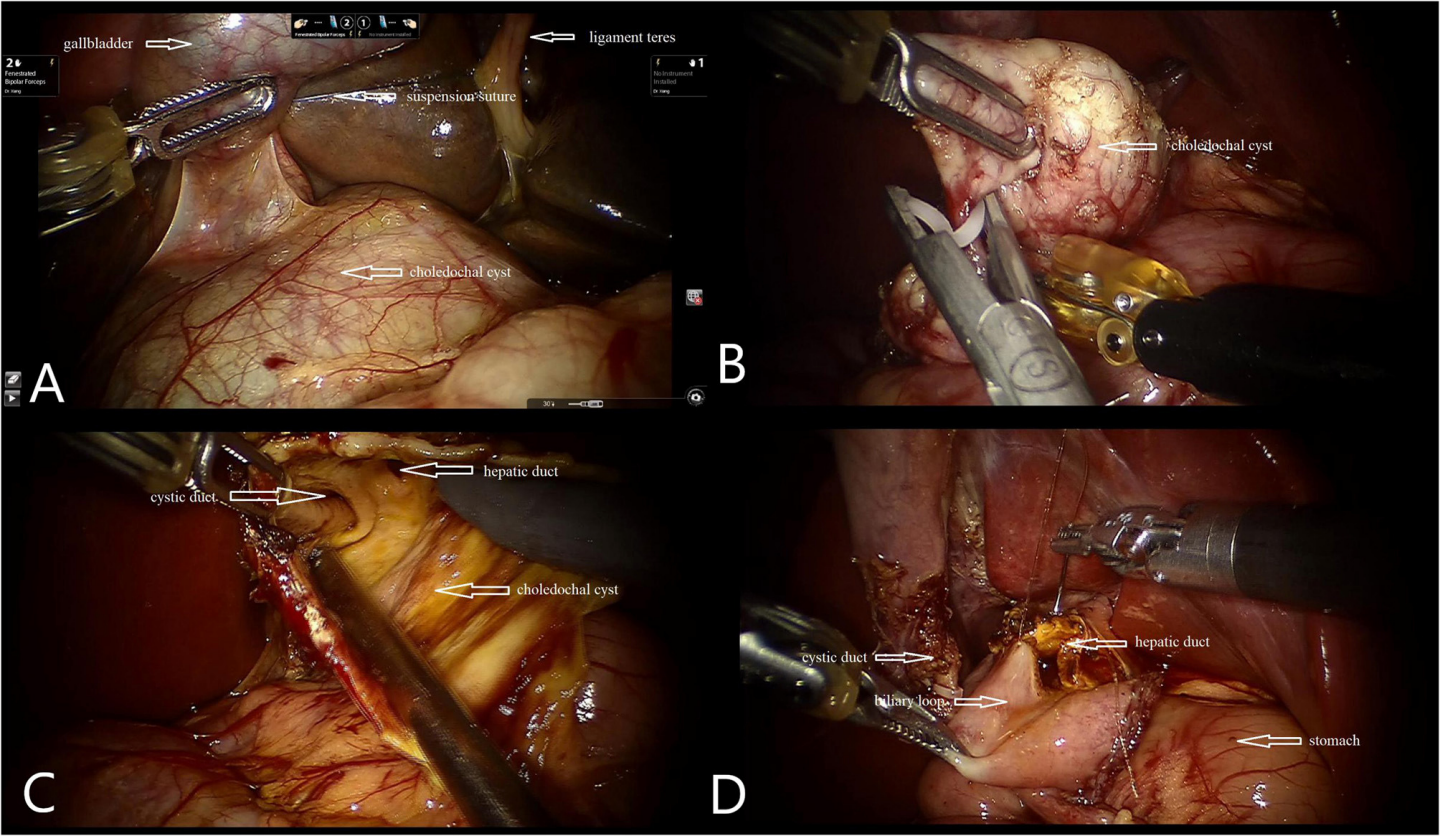
Intraoperative photographs. **(A)** Suspension of gallbladder and ligamentum teres. **(B)** Ligation of the distal end of the cyst. **(C)** Confirm cystic duct and hepatic duct. **(D)** Hepaticojejunostomy with robotic instruments.

A gastric tube was first set in place for gastrointestinal decompression after general anesthesia had been induced. The patient's right upper abdomen should be raised with his/her head elevated 15° with a left incline of 15°. End-to-side anastomosis of the jejunum was performed extracorporeally with a 1.2- to 1.5-cm incision below the umbilicus, and the intestine was returned to the abdominal cavity. A 12-mm trocar was then set as a 3D endoscopic port at the subumbilical incision and constructing pneumoperitoneum with a 12-mmHg pressure (Figure 1). With the use of images from the camera, two 8-mm trocars were placed in the right upper abdomen 5–8 cm away from the umbilicus and 4 cm below the front rib of the left axillary line. Following this, No. 1 and No. 2 arms were introduced, and a 5-mm trocar was placed between the endoscopic port and No. 1 arm as an auxiliary port. The ligamentum teres hepatis and the middle part of the gallbladder were suspended with a 3-0 sliding line to expose the cyst and hilar (Figure 2A). If the cyst was large, decompression was performed first. The anterior and posterior walls of the cyst were dissected using an electric hook close to the cyst wall. The distal end would then be dissected to the proximal pancreaticobiliary junction, and the distal end ligated with a 5-0 synthetic clip (JY1004-2103005; Zhejiang Wedu Medical; No. 3766, South Circular Road, Binjiang District, Hangzhou, China) (Figure 2B). This led to the dissection of the triangle of the gallbladder and ligation of the cystic artery and cystic duct. The proximal end of the cyst was dissected in reverse along the cyst wall to the hepatic duct of the hilar part and was removed (Figure 2C). After this, the biliary loop was lifted up to the hepatic hilum through the right mesentery of transverse colon. A 4-0 StratafixTM (SXMD1B402; Surgical Specialties Corporation; 247 Station Drive, NE1 Westwood, MA, USA) was used for end-to-side choledochojejunostomy. The anastomosis was performed from the posterior wall to the anterior wall and from the right side to the left side of the child (Figure 2D). The transverse mesocolonic hiatus was closed with a 3-0 absorbable suture, and the gallbladder was removed with the electric hook. Wrapping up the operation, a drainage tube was placed around the anastomotic site.

### Intraoperative and Postoperative Observations and Recording Indicators

A liquid diet was initiated following the recovery of intestinal function. Discharge would only be arranged when a patient could eat normally without abdominal pain or any other discomfort. Demographic information was monitored and recorded, as well as clinical manifestation, cyst type, diameter of cyst, operation time, anesthesia time, intraoperative bleeding, transfusion, time to taking water, hospital stay, and postoperative complications.

### Statistical Analyses

The statistical data were entered into Excel 2007 and analyzed using SPSS 23.0 software. The numerical variables were expressed by both the mean and standard deviation, and the categorical variables were expressed by counts (N) and percentage (%).

## Results

Ten patients below the age of 1 year and diagnosed with choledochal cysts were treated with robot-assisted procedures and subsequently analyzed in our study. The average follow-up time was 24 months. The baseline data of the 10 children are shown in Table 1. Three of the patients were male and two were female. The children's average age was 8.5 months, and the average weight was 9.11 kg. About 50% of these patients suffered from abdominal pain, while 30% experienced vomiting and 10% jaundice. Palpable abdominal masses were observed in 40% of the patients. Eight of them were Todani type Ia, and two were Todani type Ic. The average diameter of the choledochal cyst was 4.26 cm.

**Table 1:** Characteristics of the patients.

***N* = 10**	**Sex (M/F)**	**Age** **(month)^a^**	**Weight** **(Kg)^b^**	**Abdominal** **pain**	**Vomiting**	**Distension**	**Jaundice**	**Palpable** **mass**	**WBC** **(/mm^**3**^)^b^**	**N (%)^b^**	**ALT (IU/L)^b^**	**AST** **(IU/L)^b^**	**TBIL** **(umol/l)^b^**	**DBIL** **(umol/l)^b^**	**IBIL** **(umol/l)^b^**	**Cyst** **type**	**Diameter of** **cyst (cm)^b^**
Case 1	Female	6	8.3	No	No	No	No	No	9.82	25.6	38	41	23.3	11.6	11.7	Ia	4
Case 2	Male	8	8.9	Yes	Yes	No	No	No	12.19	17.3	39	56	33.4	28.1	5.3	Ia	4.5
Case 3	Male	8	8.5	No	No	No	No	Yes	5.58	33.3	15	33	7.7	2.6	5.1	Ia	6.4
Case 4	Female	9	9.5	Yes	No	No	Yes	Yes	7.14	33.7	11	29	2.8	1.3	1.5	Ia	5.5
Case 5	Female	10	9.9	Yes	No	No	Yes	No	8.9	22.8	39	86	13.4	18.1	5.3	Ia	3.9
Case 6	Female	11	9.7	No	Yes	No	No	Yes	11.38	17.6	52	70	14	3.8	11.2	Ia	5.8
Case 7	Female	11	9.8	No	No	No	No	No	10.88	29.9	19	29	3	1	2	Ic	1.5
Case 8	Female	7	8.5	Yes	No	No	No	No	6.79	25.4	33	34	7.6	2.9	4.7	Ia	2.5
Case 9	Male	8	8.8	No	Yes	Yes	No	No	7.16	24.1	36	41	10.1	3.1	7	Ic	2.1
Case 10	Female	10	9.2	Yes	No	No	No	Yes	6.37	28.1	16	33	2.4	1.4	1	Ia	6.4
*N* = 10	3/7	8.50 (7.75–10.25)	9.11 (0.59)	5 (50%)	3 (30%)	1 (10%)	2 (20%)	4 (40%)	8.62 (2.33)	25.78 (5.70)	29.80 (13.57)	45.20 (19.38)	11.77 (9.95)	7.39 (8.13)	5.48 (3.69)	8/2	4.26 (1.79)

a *Median, interquartile range;*

b *mean, standard deviation. WBC,White blood cell count; N,Neutrophils; ALT, Alanine transferase; AST, Aspartic aminotransferase; TBIL, Total bilirubin; DBIL, Direct bilirubin; IBIL, Indirect bilirubin*.

Perioperative details and postoperative outcomes are presented in Table 2. The total average operation time was 219.5 min. The average docking time was 15.6 min, and the console time was 178.5 min. The average volume of blood loss was 17 ml. None of the 10 patients received blood transfusions or conversions. The average time of their fluid diet was 3.28 days and solid diet 3.76 days. Meanwhile, the average length of hospital stay was 7.6 days. Only one patient developed an incomplete intestinal obstruction following the operation, and this was dealt with using conservative treatment. All 10 patients eventually recovered and were discharged.

**Table 2:** Intraoperative and postoperative outcomes and complications.

***N* = 10**	**Operation time (min)^a^**	**Docking time**	**Console time**	**Anesthesia time (min)^a^**	**Intraoperative bleeding (mL)^a^**	**Transfusion rate^a^**	**Conversion to open surgery**	**Time to taking water (days)^a^**	**Time to starting solids diet (days)^a^**	**Complications**	**Hospital stay (days)^a^**
Case 1	219	17	175	244	20	0	0	3	3.7	/	7.4
Case 2	222	17	178	252	10	0	0	3.1	3.8	/	7.1
Case 3	213	15	169	234	20	0	0	3.2	3.5	/	8
Case 4	219	15	180	250	25	0	0	3.4	3.6	/	7.2
Case 5	195	16	161	236	10	0	0	3.3	3.5	/	8
Case 6	219	16	176	252	20	0	0	3.7	4	/	7.3
Case 7	212	17	177	247	20	0	0	3.5	3.7	/	7
Case 8	233	15	189	267	15	0	0	2.9	3.9	/	6
Case 9	228	14	187	260	10	0	0	3.5	3.9	Intestinal obstruction	1
Case 10	235	14	193	259	20	0	0	3.2	4	/	8
*N* = 10	219.50 (11.55)	15.60 (1.17)	178.50 (9.50)	250.10 (10.41)	17.00 (5.38)	0 (0.00%)	0 (0.00%)	3.28 (0.25)	3.76 (0.19)	1 (10.00%)	7.60 (1.04)

a *mean, standard deviation*.

## Discussion

Our team has previously published papers regarding our experiences with total robot-assisted resections of choledochal cysts in children (10). The purpose of this article is to summarize and share our valuable experience with robot surgery for choledochal cysts in children below the age of 1 year. At present, the main methods of surgery for choledochal cysts include the open approach, laparoscopic approach, and robotic approach. Laparoscopic procedures and robot-assisted procedures are both minimally invasive and thus have the advantage of being more cosmetic, leading to a faster recovery and providing a better view of deep anatomical structures such as the bile duct, portal vein, and hepatic artery than open procedures (11). However, laparoscopic surgery has not been widely used thus far because of its high technical requirements, and this is especially true for the laparoscopic hepaticojejunostomy. A steep learning curve is needed in the initial stage due to the limited operation space, limited movement of the operating instruments, and instability of the two-dimensional imaging platform. However, along with the progress of surgical technology and increased experience with this procedure, the possibility of conversion to open surgery and postoperative complications is significantly reduced. When compared with laparoscopic surgery, robotic surgery has some obvious advantages (12). Firstly, a 3D three-dimensional field of vision with magnification of up to 10 times can reveal the deep anatomical structure more clearly. In addition to this, surgeons can adjust the depth and angle of the lens according to their own preference and requirements (13). Secondly, the da Vinci robotic surgery system can completely remove the choledochal cyst to its maximum extent, from the pancreaticobiliary junction to the hilar bile duct. Moreover, the da Vinci robot surgery system's simulation manipulator is highly flexible and can simulate the translation, bending, opening, closing, rotation, and other operations of the human hand (14). It can even rotate 540° to accurately grasp, free, cut, and sew all while eliminating the problem of shakiness and providing advanced motion calibration (15). Altogether, the advantages of robotic surgery significantly reduce the difficulty of surgery. Of course, robotic surgery is not without its drawbacks though. Firstly, generally speaking, the cost of robotic procedures can be prohibitive, as it is significantly higher than that of other techniques. Yoon et al. reported that the total hospital and operation charges of robotic surgery are about 1,000 USD higher than those of laparoscopic surgery. However, making matters even worse, the patient's actual bill for robotic surgery is 4,000 USD higher than that of laparoscopic surgery (16). Moreover, in a country such as China, for example, the cost of robotic surgery rises by 20,000–40,000 RMB (the equivalent of roughly 3,000–6,000 USD) compared with open and laparoscopic methods, as evidenced from our hospital's experiences. Further making matters more complicated, the da Vinci surgical system does not allow for tactile feedback; that is, the operator cannot directly feel the mechanical response when separating, suturing, or knotting. However, it is hoped that with the overcoming of the learning curve, visual feedback through hand–eye coordination can make up for this lack of tactile feedback.

Kim et al. reported on one patient and Alizai et al. reported on five patients who after initially undergoing robotic procedures were converted to open procedures shortly thereafter (9, 17). Although the patients in our study are younger than those reported in these studies, we found no similar experience of such conversion to open surgery. Through our preliminary experiences, there are some measures that can be taken to maximize the working space in these younger and smaller children, which we would like to make clear in this following section, as it would allow for smoother surgery. (1) Creating an incision below the umbilicus offered a better visual field of the cyst than from above the umbilicus. And a 1.5-cm subumbilical incision provided enough space to perform intestinal anastomosis extracorporeally and place the camera. Port I and port II were placed in the right upper abdomen at least 5–8 cm away from the umbilicus and 4 cm below the front rib of the left axillary line. And the position of the assistant port was on the triangle diagonal line with the cyst as the apex with the 3D camera port and port II forming the bottom line. The assistant port was lower than the umbilical plane to reduce any interference with the camera port and port II (No. 2 arm). The 3D camera port and operative ports only need to be inserted into a few millimeter port to maximize the working space between the head of scope and the operating area. (2) The cysts found in patients who were <1 year old were frequently large, and cyst decompression could provide sufficient operating space. (3) In the end, it is suggested to remove the gallbladder because the middle part of the gallbladder should be suspended to allow for a clear visual field. Besides, it is not so suitable to remove the gallbladder from the abdominal cavity during operation after coming into contact with the machine.

In addition to our tips for increasing the operating space as much as possible, we would also like to offer some additional advice to ensure that the operation runs smoothly. (1) It is strongly suggested that the cyst be free as a whole without being transected. Dissect the anterior side of the cyst first and then the distal part of the cyst to the pancreatic segment close to the wall afterwards. After distal ligation, the posterior wall of the cyst can be dissected in reverse, and the direction and dissociation should be from the lower side of cyst to the upper side. (2) It needs to be remembered that there is a learning curve in robot-assisted choledochal cyst resection. Dealing with older patients is recommended in the early stages of one's operation of this procedure to become more familiar and confident with it. Then, with the accumulation of experience and the flattening of the learning curve, the age of patient being operated on could be gradually lowered. In our study, the youngest patient was 6 months old. Our initial experience suggested that robotic surgery was not recommended in children under 6 months old for safety, chiefly due to lack of space and maneuverability. Along with improvements in the area of prenatal diagnosis though, there are more reports of the discovery of prenatal choledochal cysts (18, 19). However, there is still some controversy regarding the timing of surgery in this particular group of patients (19). On the one hand, one has to consider the gestational age, comorbidity of the patient, and the difficulty in performing complex reconstructive operations in infants (20). On the other hand, early surgery is advocated in view of the risks posed by the increase in size of the cyst, inflammation, or even ruptures while under observation (21). Our experience was that children under the age of 6 months with severe inflammation, severe liver injury, or a risk of perforation with a choledochal cyst can be operated on through open surgery or even traditional laparoscopy. (3) We used three ports to complete the operation without the need for a fourth port. Specifically, we made use of the camera port, ports 1 and 2, and ultimately completed the operation with the help of an assistant port. It is very important to cultivate a skilled team to prevent any complications potentially caused by robotic instruments, and a skilled assistant can ensure the successful completion of the operation and monitor the robot arm during the operation to avoid any injury to patients. During the cyst dissection process, the assistant can use wave forceps to form tension and expose the cyst and surrounding tissues. This is done so that the chief surgeon can dissect the free cyst with the No. 1 arm electric hook. If there is bleeding affecting the visual field, the assistant can then use the suction device alternately through the assistant port to suck up the blood to ensure a clear field of vision. Likewise, during a hepaticojejunostomy, the assistant mainly lifts the Stratafix with curved pliers through the assistant port to expose the visual field, so that the chief surgeon can perform a hepaticojejunostomy using No. 1 and No. 2 arms. And if intestinal fluid and bile affect the field of vision, the assistant can also use the suction device to suck up this intestinal fluid and bile. The whole process requires skilled cooperation and understanding between the assistant and chief surgeon.

However, our study also has some limitations. Firstly, the study includes a relatively small amount of samples of children below the age of 1 undergoing choledochal cyst resection using the da Vinci surgical system. Secondly, our study is a retrospective study. Multicenter and long-term follow-up data are needed to demonstrate the true benefits of robotic surgery for treating choledochal cysts in children below the age of 1. However, our overall experience thus far has found that robot-assisted choledochal cyst excisions in patients under 1 year old is safe and feasible in pediatrics.

## Conclusion

The da Vinci surgical system is safe and feasible in the treatment of choledochal cysts in children below the age of 1.

## Data Availability Statement

The original contributions presented in the study are included in the article/supplementary material, further inquiries can be directed to the corresponding author/s.

## Ethics Statement

The studies involving human participants were reviewed and approved by the West China Hospital of Sichuan University Ethics Committee (# 1,082). Written informed consent to participate in this study was provided by the participants' legal guardian/next of kin. Written informed consent was obtained from the minor(s)' legal guardian/next of kin for the publication of any potentially identifiable images or data included in this article.

## Author Contributions

BX and JC: study conception and design, analysis and data interpretation, and critical revision. XX: study concept and design, data analysis, drafting of the manuscript, and critical revision of the manuscript. YW: study conception, data collection, and analysis. KL: data collection, conducting a research, and investigation process. CA and QW: data collection, scrub data, and maintaining research data. CW: data collection. All authors contributed to the article and approved the submitted version.

## Funding

This research was supported by the Scientific Research Starting Foundation for Introduced Talents (to JC), Major Project of Sichuan Science and Technology Department (2020YFS0108), and 1·3·5 project for disciplines of excellence, West China Hospital, Sichuan University (Project nos. ZYJC18003 and 2021HXFH020).

## Conflict of Interest

The authors declare that the research was conducted in the absence of any commercial or financial relationships that could be construed as a potential conflict of interest.

## Publisher's Note

All claims expressed in this article are solely those of the authors and do not necessarily represent those of their affiliated organizations, or those of the publisher, the editors and the reviewers. Any product that may be evaluated in this article, or claim that may be made by its manufacturer, is not guaranteed or endorsed by the publisher.
